# RNA Profiling of Mouse Ependymal Cells after Spinal Cord Injury Identifies the Oncostatin Pathway as a Potential Key Regulator of Spinal Cord Stem Cell Fate

**DOI:** 10.3390/cells10123332

**Published:** 2021-11-27

**Authors:** Robert Chevreau, Hussein Ghazale, Chantal Ripoll, Chaima Chalfouh, Quentin Delarue, Anne Laure Hemonnot-Girard, Daria Mamaeva, Helene Hirbec, Bernard Rothhut, Shalaka Wahane, Florence Evelyne Perrin, Harun Najib Noristani, Nicolas Guerout, Jean Philippe Hugnot

**Affiliations:** 1 Institut de Génomique Fonctionnelle, Université de Montpellier, CNRS, INSERM, 34295 Montpellier, France; robert.chevreau@igf.cnrs.fr (R.C.); hussein.ghazale@sri.utoronto.ca (H.G.); chantal.ripoll@igf.cnrs.fr (C.R.); anne-laure.hemonnot@igf.cnrs.fr (A.L.H.-G.); helene.hirbec@igf.cnrs.fr (H.H.); bernard.rothhut@inserm.fr (B.R.); 2 EA3830 GRHV, Institute for Research and Innovation in Biomedicine (IRIB), Normandie Université, UNIROUEN, 76000 Rouen, France; chaima.chalfouh@etu.univ-rouen.fr (C.C.); quentin.delarue@univ-rouen.fr (Q.D.); nicolas.guerout@univ-rouen.fr (N.G.); 3 Institut des Neurosciences de Montpellier, Université de Montpellier, INSERM, 34295 Montpellier, France; daria.mamaeva@inserm.fr; 4 Departments of Neurobiology and Neurosurgery, David Geffen School of Medicine, University of California, Los Angeles, CA 90095, USA; swahane@mednet.ucla.edu; 5 Department of Biology, University of Montpellier, INSERM MMDN, EPHE, 34295 Montpellier, France; florence.perrin@umontpellier.fr; 6 Institut Universitaire de France (IUF), 75231 Paris, France; 7 Shriners Hospitals Pediatric Research Center and Center for Neural Repair, Lewis Katz School of Medicine, Temple University, Philadelphia, PA 19140, USA; harun.noristani@temple.edu

**Keywords:** stem cells, spinal cord, injury, regeneration, ependyma, microglia, oncostatin

## Abstract

Ependymal cells reside in the adult spinal cord and display stem cell properties in vitro. They proliferate after spinal cord injury and produce neurons in lower vertebrates but predominantly astrocytes in mammals. The mechanisms underlying this glial-biased differentiation remain ill-defined. We addressed this issue by generating a molecular resource through RNA profiling of ependymal cells before and after injury. We found that these cells activate STAT3 and ERK/MAPK signaling post injury and downregulate cilia-associated genes and FOXJ1, a central transcription factor in ciliogenesis. Conversely, they upregulate 510 genes, seven of them more than 20-fold, namely Crym, Ecm1, Ifi202b, Nupr1, Rbp1, Thbs2 and Osmr—the receptor for oncostatin, a microglia-specific cytokine which too is strongly upregulated after injury. We studied the regulation and role of Osmr using neurospheres derived from the adult spinal cord. We found that oncostatin induced strong Osmr and p-STAT3 expression in these cells which is associated with reduction of proliferation and promotion of astrocytic versus oligodendrocytic differentiation. Microglial cells are apposed to ependymal cells in vivo and co-culture experiments showed that these cells upregulate Osmr in neurosphere cultures. Collectively, these results support the notion that microglial cells and Osmr/Oncostatin pathway may regulate the astrocytic fate of ependymal cells in spinal cord injury.

## 1. Introduction

The spinal cord lies in the caudal part of the central nervous system and conveys motor information to the muscles and relays sensory signals back to the brain. It is affected by several pathologies such as multiple sclerosis, motoneuron degeneration and traumatic injuries. No curative treatments for these diseases exist. Animals like salamanders and zebrafish regenerate spinal cord cells, including neurons, after lesions [1]. This extraordinary property is due to the persistence of fetal-like radial-glia stem cells around the adult spinal cord central canal. These cells express the FOXJ1 transcription factor and maintain the expression of spinal cord developmental genes such as Pax6 and Shh. Similar FOXJ1^+^ cells exist in mammals, including young humans, constituting the ependymal region [2,3,4,5,6]. We and others have shown that this region is organized like the germinative cell layer (namely the neuroepithelium) which generates most spinal cord cells during development. Key spinal cord developmental transcription factors and genes such as Arx, Msx1, Nestin, Pax6 and Zeb1 remain expressed in this area both in mouse and human [6].

Mouse ependymal cells are mostly CD24^+^ CD133^+^ bi-ciliated cells resembling E2 ependymal cells found in the brain lateral ventricles [7]. Compared to brain multiciliated E1 ependymocytes, spinal cord ependymal cells are enriched in genes related to the regulation of precursor cell proliferation and oligodendrocyte differentiation [8,9]. These cells also have a permissive chromatin state favoring gene expression involved in oligodendrogenesis after injury [10]. Spinal cord ependymal cells hardly proliferate in the intact spinal cord. However, it is known since 1962 [11] that after spinal cord injury (SCI) these cells rapidly proliferate via Ras signaling activation [12] and then migrate to the lesion site. This is notably observed when the lesion compromises the integrity of the ependymal region [13]. Unfortunately, they produce no neurons, generate only few oligodendrocytes, and mainly form astrocytes [14]. It was shown using genetic lineage-tracing tools that these astrocytes contribute to the core of the glial scar [15]. These ependymal cells-derived astrocytes are however beneficial for recovery, as further axonal loss and secondary enlargement of the lesion volume are observed after their ablation with genetic tools [12]. Positive effects of these astrocytes appear to be mediated by the release of neurotrophic factors, such as HGF, CNTF and IGF-1 [12].

In vitro, a fraction of these ependymal cells form neurospheres which can be propagated for several passages and then induced to differentiate into neurons and glial cells [16]. This shows that at least some ependymal cells behave as multipotent spinal cord stem cells and depending on the context, can form new neurons or glia. Importantly, when grafted in a neurogenic environment (hippocampus), cultured spinal cord stem cells can generate neurons [17]. The regenerative capacity of these cells has been recently further illustrated by showing that expression of a single oligodendrogenic transcription factor (Olig2) was enough to reprogram them to replace large numbers of lost oligodendrocytes in the injured mouse spinal cord [10].

Theoretically, such plastic cells represent an attractive endogenous source to alleviate various spinal cord lesions if one could control their proliferation and fate to produce new neurons and oligodendrocytes. However, the molecular events taking place in these cells after SCI remain largely unknown. This lack of knowledge makes it difficult to control these cells. Notably, we need to know: which genes are modulated in ependymal cells after injury? which signaling pathway is activated? what is the influence of parenchymal spinal cord cells, notably microglia, on the ependymal cell fate? why mouse ependymal cells mainly generate astrocytes after lesion? The present work was designed to provide new data on these pending issues.

Here we determined the RNA profiling of mouse ependymal cells before and after lesion. Ependymal cells are difficult to purify in vitro and their enzymatic dissociation could lead to artifactual RNA variations. To overcome this obstacle, the ependymal region of unfixed spinal cord sections was directly microdissected with a laser. This led us to uncover the implication of several signaling pathways and genes. We used immunofluorescence and cell culture to study these results further in vitro. Notably we discovered a role for the oncostatin pathway and microglia in regulating ependymal cell proliferation and differentiation. This new molecular resource and new knowledge will help to better understand ependymal cell response following injury.

## 2. Materials and Methods

### 2.1. Animals

Animals were handled at the animal care facility in compliance with the Committee of the National Institute of Health and Medical Research (INSERM) in accordance with the European Council directive (2010/63/UE) for the protection and use of vertebrate animals. All animals were handled under pathogen-free conditions and fed chow diet *ad libitum*. Adult CD1 mice (3 months, Charles River, Ecully, France) were used for microdissection after spinal cord injury, RNA profiling, neurosphere cultures and histology. C57BL/6 mice aged 3–4 months were used for spinal cord organotypic slice cultures and histology. CX3CR1^+/GFP^ C57BL/6 [18] Charles River) transgenic mice aged 3 months were used for spinal cord microglia histology. β-actin-GFP C57BL/6 mice [19] (Charles River) aged 3 months were used to derive GFP^+^ neurospheres to co-culture with microglial BV-2 cells. For neurosphere cultures, RNA and protein extractions, adult spinal cords were dissected from mice euthanized by intraperitoneal injection of sodium pentobarbital (100 mg/kg).

### 2.2. Cell Cultures

Neurosphere cultures were derived from adult spinal cord using the protocol and medium detailed in [20]. Neurospheres after 3 passages were used for RNA profiling and cell fate/proliferation experiments. In the growing condition, medium contained EGF, FGF2 (10 ng/mL each, Peprotech, Neuilly-sur-Seine, France) and Heparin (2 µg/mL, H3149, Sigma, Saint-Quentin-Fallavier, France). For differentiation, enzymatically-dissociated neurospheres were plated on poly-D-lysine/Laminin (1 µg/cm^2^) coated glass coverslips in a medium without growth factors and heparin but containing 2% fetal bovine serum (ThermoFisher, Illkirch, France). After 4 days, the coverslips were either fixed for immunofluorescence or processed for RNA extraction (ReliaPrep RNA Miniprep kit, Promega, Charbonnières-les-Bains, France) for microarray analysis. To test the influence of cytokines on cell growth/differentiation and OSMR expression (QPCR, Figure 5C), all cytokines (Peprotech) were used at 10 ng/mL. Influence of OSM on cell growth (Figure 5G) was measured by seeding dissociated cells (5000 cells per well, 6 wells) in 1 mL of medium using 24-well plates coated with poly-HEMA (P3932, Sigma, Saint-Quentin-Fallavier, France) to inhibit cell adherence. After 5 days, the neurospheres were directly dissociated by addition of trypsin in the wells (0.5% final) and the cell number was measured with an automated cell counter (Z2, Beckman Coulter, Villepinte, France).

To assess the influence of microglia on spinal cord neurosphere cells, BV-2 immortalized microglial cells [21] (passage 3 to 8) cultured in macrophage serum-free medium (12065-074, ThermoFisher, Illkirch, France) were used. Neurosphere cultures derived from β-actin GFP spinal cord were cultured alone or in the presence of BV-2 cells (ratio BV-2 vs spinal cord cells was 1:6) in the neurosphere medium containing growth factors. After 3 days, cultures were dissociated with trypsin/EDTA 0.25% and GFP^+^ spinal cord cells were collected by FACS for RNA extraction and QPCR.

For spinal cord organotypic slices, the protocol described in [22] was used except that glutamine (2 mM final concentration) was used instead of glutamax and poly-D-lysine instead of poly-L-lysine for coating. Slices (300 µm) were fixed with paraformaldehyde (4%, 20 min, room temperature) directly after sectioning (t = 0) or after 72 h in culture. Slices were then cryopreserved in PBS-sucrose 10, 20, 30% and embedded in OCT for cryosectioning and immunofluorescence.

### 2.3. QPCR

cDNA synthesis was performed using 1–5 μg of total RNA with random hexamers and reverse transcriptase (GoScript, Promega, Charbonnières-les-Bains, France). Quantitative RT-QPCR was performed in triplicates for each samples using the KAPA SYBR PCR kit (KK4600, Sigma, Saint-Quentin-Fallavier, France) with a LightCycler 480 apparatus (Roche, Basel, Switzerland). 10–100 ng of cDNA was used for each reaction. Primers are listed in Table S2. Relative expression values were calculated using the 2^−ΔΔCT^ method and normalized using the β-actin gene. All QPCR reactions were performed with three independent cultures.

### 2.4. Western Blot

Total proteins from spinal cord samples and cultured cells were extracted and used for Western blots as described in [23]. Proteins were detected using the Odyssey CLx Li-Cor technology (Li-Cor, Bad Homburg, Germany). Briefly, primary antibodies were incubated in Li-Cor PBS buffer overnight at 4 °C. After washing, membranes were incubated with secondary fluorescent dye (IRDye 800CW for OSMR protein and IRDye 680LT for β-actin normalization). For Figure 5D–F, signals were obtained with peroxidase-conjugated secondary antibodies (Cell Signalling Technology, Danver, MA, USA) and revealed with Clarity Western ECL kit (BioRad, Marnes-la-Coquette, France) and a ChemiDoc™ XRS Imaging system (BioRad).

### 2.5. ELISA

The presence of OSM cytokine in the medium of spinal cord neurospheres and BV-2 cells was detected by solid-phase sandwich ELISA (Quantikine mouse OSM kit, R&D, Minneapolis, MN, USA) with recommendations of the manufacturer. Absorbance measurements were done at 450 nm with a CLARIOSTAR microplate reader. 50 µL of medium conditioned by BV-2 cells or neurosphere cultures for 3 days was used for these experiments. Pure OSM supplied with the kit was used for reference curve.

### 2.6. Equipment and Settings

Fluorescent images were taken with a Zeiss apotome Axio Imager 2 (Zeiss, Paris, France) equipped with a Zeiss ZEN software. Main settings were: binning 2 × 2, apotome mode: 5.

### 2.7. Spinal Cord Injury

**Figure 1:**
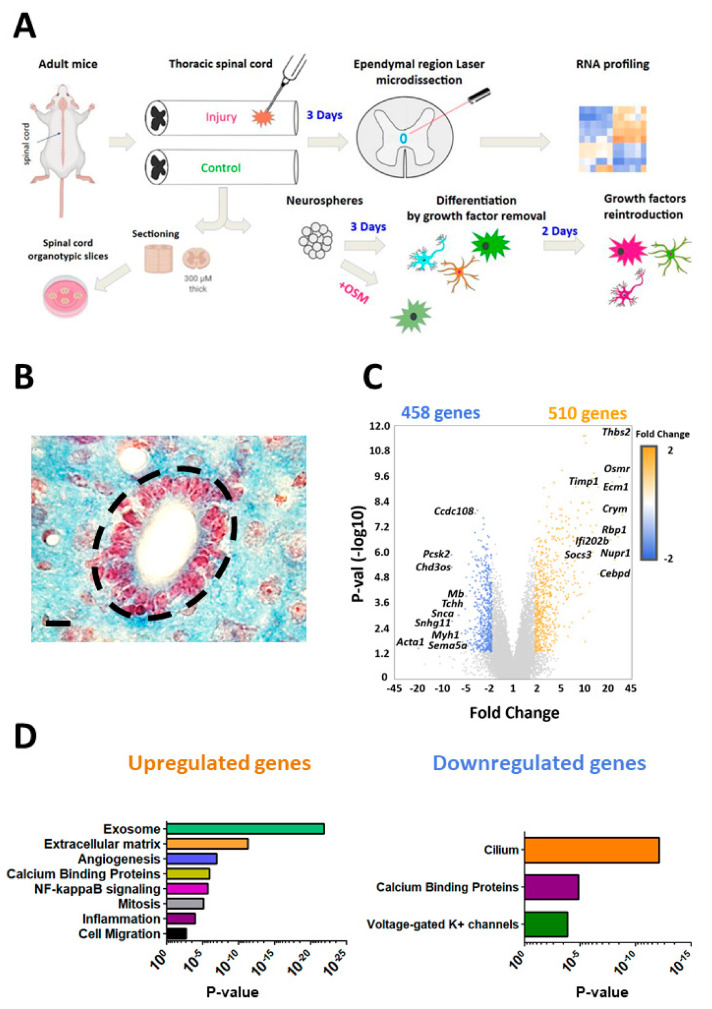
Bioinformatics analysis of ependymal gene expression in SCI. (**A**): Schematic overview of main article steps and techniques. (**B**): Mouse spinal cord ependymal region. Ependymal cell nuclei (thoraco-lumbar level) are stained in red with neutral red dye. Surrounding parenchyma is stained in blue with luxol fast blue. The microdissected area is delimited with black dashed lines. (**C**): Volcano plots of genes whose expression are dysregulated after injury in the ependymal region (fold change ≥2 or −2). Top10 up and downregulated genes are indicated. (**D**): Selection of Gene Ontology analysis for up- and down-regulated genes with *p*-values. Comprehensive gene ontology analysis is provided on Table S1.

Adult CD1 mice aged 3 months were anesthetized using isoflurane gas (1.5%, O_2_ 1 L/min). T9–T10 dorsal laminectomy was performed to expose the spinal cord. Four needle penetrations (30G) were done (staggered holes, 2 on each side of the posterior spinal vein). Penetration depth was approximately 1 mm. We chose this type of injury so as to not directly damage the ependymal region which would have made the laser microdissection difficult to achieve with accuracy and to avoid contaminations by other cells, blood and debris. This type of lesion is enough to trigger ependymal proliferation detected by EdU incorporation (data not shown) which is also confirmed by RNA profiling showing an upregulation of mitotic genes (Figure 1D). After injury, muscles and skin were sutured and animals were placed on heated pads until complete recovery. For control experiments, we performed laminectomy but no needle insertion (sham injury). To determine the number of microarrays needed to reach an acceptable statistical power (1-β), we used the online tool from the Department of Bioinformatics and Computational Biology, MD Anderson center, University of Texas (https://bioinformatics.mdanderson.org/MicroarraySampleSize/ (accessed on 1 October 2021). Calculation showed that 6 animals per group were enough to reach a statistical power of 0.8.

### 2.8. Histology and Immunofluorescence

**Figure 2:**
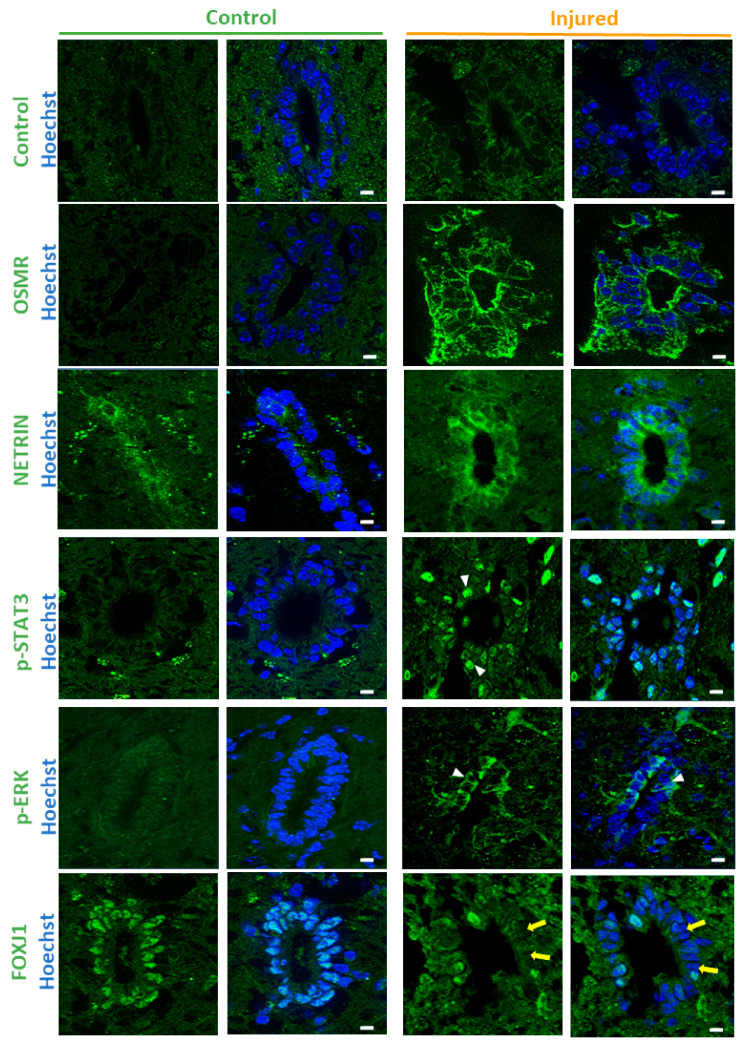
Immunofluorescence validation. Immunofluorescences performed on control and injured spinal cords for indicated proteins. Control immunofluorescence was performed using antibody against the synthetic DYKDDDDK TAG antigen. White arrowheads and yellow arrows show examples of positive and negative cells respectively. These images are representative of 2 independent experiments (*n* = 6 animals in total, 7–15 sections examined per animal). Scale bars = 10 µm.

For spinal cord histology, mice were anesthetized by intraperitoneal injection of sodium pentobarbital (100 mg/kg) and perfused intracardially with 10 mL of PBS followed by 50 mL of 4% paraformaldehyde-PBS solution (pH 7.0). After dissection, spinal cords were post-fixed in the same solution for 1 h at 4 °C and cryopreserved by successive immersion in 10%, 20%, and 30% sucrose solutions in PBS for at least 6 h. Thoracic segments of the spinal cord were cut, embedded in OCT medium, rapidly frozen in liquid N2-cooled isopentane and cryosectioned transversally (14 µm) (Leica apparatus). Immunofluorescences were performed on slide-mounted spinal cord sections with primary antibodies and dilutions listed in Table S2 after permeabilization for one hour with 0.1–0.3% Triton 100× and 5–10% donkey serum. For cell cultures, immunofluorescences were done on cells grown on poly-D-lysin/laminin coated glass-coverslips and fixed with paraformaldehyde (4%, 20 min, room temperature). Secondary antibodies (Alexa488 or Alexa594-conjugated species-specific anti-mouse, rabbit or goat) were purchased from Jackson Immunoresearch, Cambridge, UK. Incubations without primary antibody or with antibody recognizing antigens not present in the sections (monoclonal anti DYKDDDDK Tag or polyclonal anti GFP) were used as negative controls. Nuclei (blue in all images) were stained with Hoechst 1 µg/mL for 10 min. For p-STAT3 stainings, antigen retrieval was done for 25 min at 90 °C in citrate buffer pH 6.0 in a histology microwave oven (Histos 5, Milestone). The quality of stainings was evaluated by two independent investigators (JPH, HG, RC or CR). For stainings on SCI sections (Figure 2), we selected sections where the needle tract was present to make sure we were close to the injury point. Luxol fast blue and neutral red staining (Figure 1B) was performed as described in [24]. All presented images are representative images and the number of examined sections and animals are indicated in the legends.

### 2.9. RNA Extraction, RNA Profilings and Bioinformatics Analysis

Laser microdissection of the ependymal region in sham and injured spinal cord (72 h post injury, 6 animals each condition) were performed exactly as described in details in [6]. After dissection, a segment of the thoracic spinal cord containing the needle insertion sites was selected and flash frozen in N2 without chemical fixation. RNA from laser-microdissected areas and neurosphere cultures (passage 3) were extracted with a ReliaPrep RNA cell Miniprep kit (Promega, Charbonnières-les-Bains, France) according to the manufacturer’s protocol and quantified/qualified with Nanodrop and Agilent 2100 Bioanalyzer apparatus (RNA Integrity Number (RIN) >8.0). RNA labelling and profiling was performed using Affymetrix mouse microarrays as described in [6]. Gene expression data were normalized with RMA algorithm and analyzed using the Affymetrix TAC 4.0 software (Transcriptome Analysis Console, Affymetrix, Santa Clara, CA 95051, USA). The filter criteria were set to a linear fold change ≥2 or ≤−2 (control vs injured spinal cord) with *p* value ≤ 0.05. Gene lists were analyzed with DAVID Bioinformatics Resources 6.83 for gene enrichment analysis [25]. Data were also processed to search for pathway activation (Figure S1B) through the use of ingenuity pathway-analysis (IPA) software (Qiagen Inc. Courtaboeuf, France) [26].

### 2.10. Statistical Analysis and Countings

All experiments and stainings were performed at least three times and most of them were done four times. For cell countings, 6–10 independent fields distributed over 3 coverslips of the same condition were counted manually under the microscope or through image captures and the ImageJ software cell counting tools. In total, not fewer than 300 cells were counted per experiments. We selected fields where the cell density was similar as this parameter can influence the cell phenotype. Data are represented as means ± standard error of the mean (S.E.M). Statistical differences in experiments were analyzed with tests indicated in the legends using GraphPad Prism software and BootstRatio website (http://rht.iconcologia.net/stats/br/ (accessed on 1 February 2020) for QPCR [27]. Significances: *** *p* < 0.001, ** *p* < 0.01, * *p* ≤ 0.05.

## 3. Results

### 3.1. Ependymal Cell RNA Profile Is Highly Modified by SCI

We explored the mechanisms underlying ependymal region activation by performing spinal cord injury with multiple needle insertions in six mice. Six other mice with sham operation were used as control. This model mimics bullet or knife spine injuries which are observed in human [28,29] and is enough to trigger ependymal cell proliferation over the 3 days post injury in mouse (data not shown). After 3 days, the central canal region was microdissected (Figure 1A,B) and RNAs were analysed with microarrays as previously described [6]. Two distinct RNA profiles were observed in ependymal cells microdissected from control and injured spinal cords (Figure S1A).

Data analysis revealed that 510 genes were significantly upregulated (*p* ≤ 0.05; fold change ≥2) while 458 genes were downregulated (fold change ≤−2) (Figure 1C). Top upregulated and downregulated genes are shown in Table 1 and the full gene list is in Table S1. Seven genes were upregulated more than 20-fold, namely Crym, Ecm1, Ifi202b, Nupr1, Osmr, Rbp1 and Thbs2 whereas only one was downregulated to this extent (Acta1). CRYM and RBP1 are two proteins implicated in pipecolic acid and retinol metabolism respectively [30,31]. NUPR1 and IFI202B are involved in transcriptional response to various cellular stress notably by binding to P53 and by inhibiting apoptosis [32,33]. THBS2 is an adhesive glycoprotein and ECM1 is a protein of the extracellular matrix involved in angiogenesis [34]. OSMR is the receptor for the oncostatin cytokine [35] and ACTA1 is an isoform of actin found in muscle tissues and is also highly enriched in ependymal cells [6]. These modifications suggest that SCI triggers major changes in ependymal cell metabolism, transcriptional networks and phenotype.

We then submitted the dysregulated gene list to GO and pathways analyses (Figure 1D and Table S1). As expected from the well-documented proliferation of ependymal cells after SCI, many genes related to cell cycle such as Myc and Cyclins (A2, B1/2 and D1/2) were upregulated by SCI. More interestingly, RNAs related to several extracellular matrix proteins such as Fibronectin (Fn), Matrilin (Matn2), Vitronectin (Vtn), Versican (Vcan), and several Collagens (Col8A1, Col11A1, Col12A1, Col14A1) were also upregulated. Finally, ependymal cells reacted to SCI by the modification of expression of 63 genes closely related to transcription (42 and 21 up and down regulated respectively) (Table 1 and Table S1). Five were upregulated more than 10-fold (Cebpd, Etv5, Fos, Nupr1, Ifi202b). CEBPD is an important transcription factor regulating the expression of genes involved in immune and inflammatory responses [36]. ETV5 (also known as ERM) is a member of the PEA3 subfamily of ETS transcription factors. Its expression is notably controlled by the ERK/MAPK pathway and is often correlated to cell proliferation and migration [37]. Finally, FOS is a well-known proto-oncogene regulating cell proliferation and differentiation in many contexts [38].

To assess whether these RNA variations led to protein level modifications, we selected two genes: one gene which is highly overexpressed after SCI (30×): Osmr and a second gene which is only moderately increased (2.5×), Ntn, coding for NETRIN, a secreted protein involved in many processes such as stem cell renewal and apoptosis [39]. Figure 2 shows weak or no detection of these 2 proteins by immunofluorescence in the control ependymal region but a clear induction in ependymal cells after injury. This shows that the SCI-induced transcriptional modifications lead to new proteins in ependymal cells.

**Figure 3:**
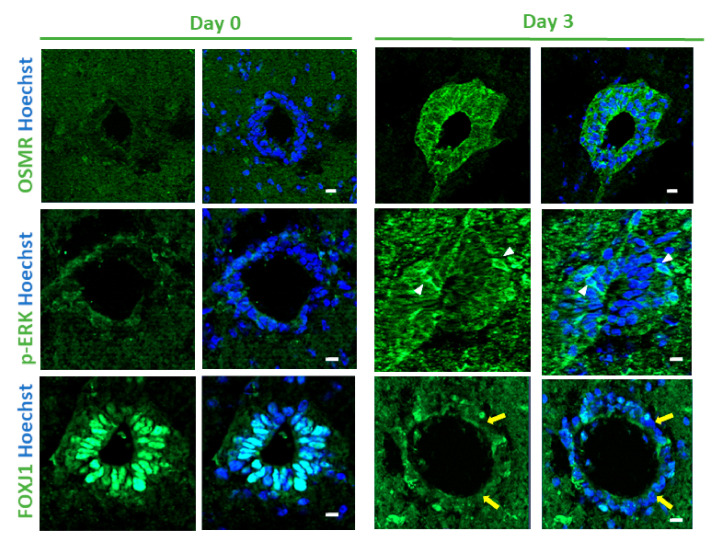
Spinal cord organotypic slice cultures. Immunofluorescences for indicated proteins performed on spinal cord slices after collection (Day 0) and after 3 days in culture (Day 3). White arrowheads and yellow arrows show examples of positive and negative cells respectively. These images are representative of 3 independent experiments. (*n* = 4 animals, 5 sections examined per culture). Scale bars = 10 µm.

To understand further the signaling pathways affected by SCI in the ependyma, we used a causal analysis approach with Ingenuity pathway application [26]. Two pathways were identified (Figure S1B): MAPK/ERK and STAT3 signalings. It was important to validate these bioinformatics predictions by performing immunofluorescences against the phosphorylated forms of STAT3 and ERK/MAPK (p-STAT3 and p-ERK) which marks activation of these proteins. Figure 2 shows that whereas we could not detect positive cells in sham animals, a fraction of cells around the central canal expressed p-STAT3 and p-ERK after SCI. We decided to support our results further by using organotypic cultures of adult spinal cord slices (Figure 1A). In this ex vivo model, spinal cord sectioning and probably culture conditions activate ependymal cell proliferation [22]. We performed immunofluorescence for p-ERK, p-STAT3 on slices after 0 and 3 days of culture. Figure 3 shows that in this model, the p-ERK protein was clearly induced in a fraction of ependymal cells after 3 days of culture. No convincing and reproducible stainings were observed for p-STAT3 (data not shown).

### 3.2. Cilia Genes Are Downregulated after SCI and Are Controlled by Growth Factors

**Figure 4:**
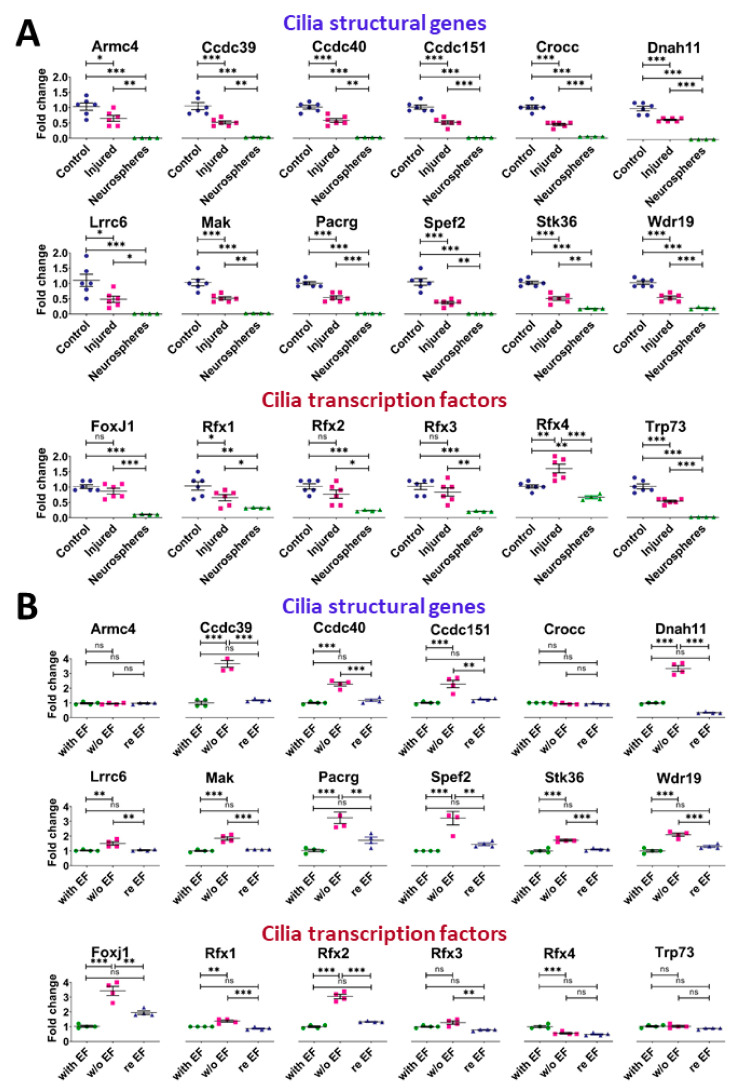
Cilia gene expression is affected by SCI and culture conditions. (**A**): Microarray quantifications of indicated genes in control vs injured spinal cord ependyma and growing spinal cord neurospheres (*n* = 6 for spinal cords and *n* = 4 for neurosphere cultures). Tests = one way ANOVA with Tukey post-hoc tests. Values represent fold changes compared to control spinal cords. (**B**): Microarray quantifications of indicated genes in neurospheres cultured in 3 conditions (*n* = 4 for each conditions): with EF (i.e., growing with EGF and FGF2), w/o EF (i.e., differentiated by removing EGF and FGF2), and re EF (reintroduction of EGF and FGF2 for 3 days after the differentiation step). Tests = one way ANOVA with Tukey post-hoc tests. Values represent fold change compared to neurospheres in the growing condition. ***, *p* < 0.001; **, *p* < 0.01; *, *p* ≤ 0.05. n.s. = not significant.

We previously reported that, consistent with their functions, ependymal cells highly expressed genes involved in ciliogenesis [6]. Cilia are key organelles not only involved in cell movement but are also major regulators of cell proliferation [40]. Most cells begin to disassemble their primary cilia at cell cycle re-entry [41] and reintroduction of cilia genes in cancer cells can lead to proliferation arrest and differentiation [42]. Remarkably, GO analysis revealed a significant downregulation of these genes after SCI (Figure 1D and Table S1) suggesting a link with the concomitant upregulation of genes involved in mitosis (Figure 1D). Very little is known about the molecular mechanisms which trigger cell proliferation of ependymal cells after SCI and we considered that studying the modification of cilia gene expression presented some relevance to this issue. To further illustrate the downregulation of cilia-related genes in SCI, we selected 12 genes (Armc4, Ccdc151, Ccdc39, Ccdc40, Crocc, Dnah11, Lrrc6, Mak, Pacrg, Spef2, Stk36, Wdr19) with documented roles in cilia and which we found specifically expressed in the spinal cord ependymal region in our previous study (enrichment fold change >16 compared to the spinal cord parenchyma [6]). Microarray analyses show that the expression of these 12 genes were significantly downregulated between 1.5- and 2.7-fold after injury (Figure 4A). This suggested that transcription factors governing ciliogenesis might be also affected by SCI. As we previously found [6] that mouse spinal cord ependymal cells specifically express several transcription factors involved in cilia formation, namely FoxJ1, Rfx1-4 [43] and Trp73 [44], we analysed their expression after SCI. Microarray results presented in Figure 4A indicated that Rfx1 and Trp73 RNA levels were significantly reduced whereas Rfx4 expression was increased. With regards to FoxJ1 and Rfx2, their expression also appeared to be reduced after SCI but did not reach significance (FoxJ1: mean ± S.E.M. = 1.01 ± 0.14 vs 0.86 ± 0.22 post-SCI and Rfx2: 1.01 ± 0.21 and 0.76 ± 0.32 post-SCI, *n* = 6). As Foxj1 is the central transcription factor orchestrating cilium morphogenesis [45] and as this protein can also be rapidly degraded by proteasome [46], we also explored its expression at the protein level by immunofluorescence. Figure 2 shows that compared to control spinal cord, FOXJ1 expression is reduced in ependymal cells after SCI and some of them became negative. We validated this downregulation further using the spinal cord organotypic slice model mentioned previously (Figure 1A). Figure 3 shows that, compared to control slices, FOXJ1 expression was barely detected in ependymal cells maintained for 3 days in culture.

Next, we wanted to get an insight on the molecular mechanisms underlying the downregulation of cilia-related genes in spinal cord ependymal cells. We used spinal cord-derived neurospheres as an in vitro model. These neurospheres originated from the central canal ependymal cells [15] and are composed of progenitor/stem cells [47]. We derived four independent neurosphere cultures from adult spinal cords and performed RNA profiling of these cells after 3 passages using microarrays. Data presented in Figure 4A indicates that neurosphere cells expressed the 12 selected cilia genes and six cilia transcription factors (FoxJ1, Rfx1-4, Trp73) at a considerably lower level than in vivo ependymal cells.

**Figure 5:**
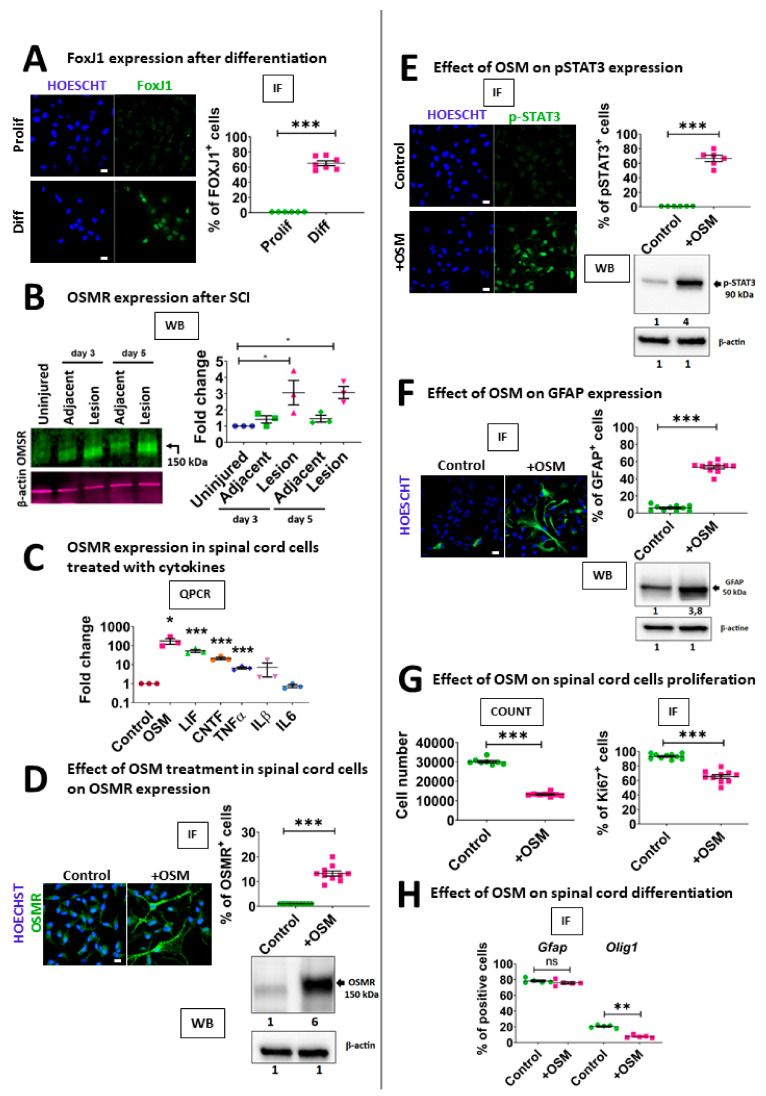
OSM affects OSMR expression, neurosphere growth and differentiation. (**A**): Left-hand images: immunofluorescences for FOXJ1 in spinal cord neurosphere cells cultured in proliferation (with growth factors) or differentiation (without growth factors) conditions. Scale bar = 10 µm. Right panels: immunofluorescence quantification (*n* = 7 fields). (**B**): Left-hand panel: WB for OSMR on proteins extracted from control and injured total spinal cord (3 and 5 days post injury). Proteins were extracted from spinal cord segments containing and adjacent to the lesion site. β-actin was used for normalization. Right-hand panel: WB quantification. Values represent fold change compared to uninjured spinal cord protein extracts. Tests = one way ANOVA with Tukey post-hoc tests. *n* = 3 independent experiments. (**C**): QPCR for Osmr RNA in growing spinal cord neurospheres treated for 3 days with indicated cytokines. Values represent fold change compared to untreated neurospheres. Statistical tests were performed with Bootstratio [23] compared to non-treated neurospheres. *n* = 3 independent experiments. (**D**): Left-hand panels: immunofluorescences for OSMR in control or OSM-treated spinal cord neurosphere cells. Scale-bar = 10 µm. Right-hand panel: immunofluorescence quantification (*n* = 10 fields). Test = two tailed *t*-test. Lower panel. WB for OSMR on proteins extracted from untreated and OSM-treated neurospheres. Numbers represent quantification (fold change) compared to untreated neurospheres. β-actin was used for normalization. (**E**): Left-hand panels: immunofluorescences for p-STAT3 (phospho-STAT3) in control or OSM-treated spinal cord neurosphere cells. Scale-bar = 10 µm. Right-hand panel: immunofluorescence quantification (*n* = 6 fields). Test = two tailed *t*-test. Lower panel. WB for p-STAT3 on proteins extracted from untreated and OSM-treated neurospheres. Numbers represent quantification (fold change) compared to untreated neurospheres. β-actin is used for normalization. (**F**): Left panels: Immunofluorescence for GFAP in untreated and OSM-treated neurosphere cell cultures. Right-hand panel: immunofluorescence quantification (*n* = 10 fields). Test = two tailed *t*-test. Lower panel: WB for GFAP on proteins extracted from untreated and OSM-treated neurospheres. Numbers represent quantification (fold change) compared to untreated neurospheres. β-actin is used for normalization. (**G**): Effect of OSM on neurosphere growth. Left-hand panel: Diagram shows the number of cells obtained in untreated and OSM-treated neurospheres 6 days after seeding (*n* = 8 wells). Test = two tailed *t*-test. Right-hand panel: Diagram shows the % of MKI67^+^ cells obtained in untreated and OSM-treated cell cultures (*n* = 10 fields). Test = two tailed *t*-test. (**H**): Effect of OSM on spinal cord stem cell differentiation. Diagram shows the % of GFAP^+^ and OLIG1^+^ cells, 4 days after differentiation in untreated and OSM-treated cell cultures. Tests = Mann-Whitney tests, *n* = 5 independent experiments. ***, *p* < 0.001; **, *p* < 0.01; *, *p* ≤ 0.05. n.s. = not significant. IF = immunofluorescence.

Compared to ependymal cells which proliferate at a very low rate, neurosphere cells are highly proliferative with a doubling time of approximately 28 h (unpublished data). Considering the low expression of cilia genes found in neuropheres (Figure 4A), we hypothesized that these genes might be negatively controlled by growth factors. We tested this possibility by removing the growth factors (EGF/FGF2) in the medium for 3 days and then by monitoring gene expression with microarrays (*n* = 4). Indeed, Figure 4B shows that growth factor removal (w/o EF condition Figure 4B) significantly increased the expression of 12/14 cilia-related genes (fold change between 1.5 to 3.9) together with 3 transcription factors (FoxJ1, Rfx1, Rfx2) (fold change 3.4, 1.3 and 3.1, respectively). Conversely Rfx4 was downregulated. We validated this result at the protein level for FOXJ1. Indeed, whereas FOXJ1 is not detected by immunofluorescence in neurosphere cells cultured with growth factors, it is well-expressed after growth factor removal (Figure 5A). To further test the influence of growth factors on the expression of these cilia-related genes, we reintroduced them in the medium for 3 days (re EF condition Figure 4B) and performed new microarray analysis (*n* = 4). This led to a significant downregulation of 12/14 cilia-related genes together with FoxJ1, Rfx1 and Rfx2 (Figure 4B). This illustrates the negative influence of growth factors on cilia-related genes and cilia transcription factors in neurosphere cells. Collectively, these results support the notion that SCI opposes cilia-related gene expression in ependymal cells, possibly by inducing proliferation and downregulating the expression of FOXJ1 transcription factor.

### 3.3. Oncostatin Affects Proliferation and Differentiation of Spinal Cord Neurosphere Cells

Besides proliferation, long term genetic-lineage tracing experiments have shown that ependymal cells mostly differentiate into astrocytes after injury [14]. In line with these observations, our RNA profiling revealed that GFAP, a cytoskeleton gene typically expressed by astrocytes was upregulated 12.9-fold (Table 1) in ependymal cells after SCI. Four other commonly used markers for astrocytes (ALDH1L1, ALDOC, FABP7, VIM) were also significantly increased (fold changes respectively: 4.5; 3.1; 3.7; 2.1; Table S1). In contrast, four markers (CCDC153, HDC, ODF3B, TMEM212) specific for ependymocytes according to the PanglaoDB database and highly expressed in the mouse central canal region [6], were significantly downregulated (fold changes respectively: −3.7; −2.6; −2.6; −2.6; Table S1). This suggested that ependymal cells rapidly engage into astrogenesis as early as within 3 days after injury. To get insight on the molecular mechanisms underlying this differentiation, we examined the list of dysregulated genes for possible candidates. The second most upregulated gene (×30) was the receptor for oncostatin (OSMR) and we selected this gene for further investigations as (i) oncostatin is a member of the Il-6 cytokine family and these cytokines are well-known to be strong inducers of astrocytic differentiation of neural stem cells though STAT3 activation [48] (ii) OSMR is expressed by GFAP^+^ astrocytes in the adult brain subventricular zone and in the olfactory bulb [49,50].

We started our investigation by further validating the sharp upregulation of the OSMR protein we observed in ependymal cells (Figure 2) using the spinal cord organotypic slice model we described above (Figure 1A). Figure 3 shows that after 3 days of culture, ependymal cells highly expressed OSMR detected by immunofluorescence. We also performed western blot using proteins extracted from uninjured total spinal cord and 3 and 5 days after SCI. This again revealed a sharp induction of OSMR by SCI (Figure 5B). It is worth noting that here, the detection of OSMR reflects its induction in ependymal cells but probably also in other undefined cells in the spinal cord parenchyma which may overexpress OSMR after SCI.

We then sought to understand what triggers the sharp OSMR upregulation after SCI in ependymal cells. It has been shown that Omsr is induced by various cytokines, notably belonging to the IL-6 family, in different cell types. To evaluate this possibility in the spinal cord context, we used again the neurosphere model (Figure 1A). Spinal cord neurospheres cultured in the proliferating condition with growth factors were treated for 5 days with IL-6 related cytokines (CNTF, IL-6, LIF, OSM), inflammatory cytokines (Interferon𝛾, CCL2, CXCL3, TGFβ, TNFα) and various cytokines which have been shown to influence neural precursor fate (GDF15, VEGFC, BMP6). The effect on Osmr expression was measured by QPCR. We found that only four of these cytokines (CNTF, LIF, OSM and TNFα) upregulated Osmr expression (Figure 5C). OSM, LIF and CNTF were particularly potent with 175-, 50-, and 20-fold increase respectively (Figure 5C). Western blot and immunofluorescence also validated the strong increase of OSMR in neurosphere cells after OSM treatment (Figure 5D). In the brain, these IL-6 related cytokines can activate STAT3 signaling in neural stem cells that is accompanied by proliferation reduction and astrocytic differentiation [48]. Indeed, we found that addition of OSM to growing neurospheres induced a sharp increase of p-STAT3 expression (Figure 5E) and astrocytic differentiation as revealed by immunofluorescence and western blot for GFAP (Figure 5F). OSM also negatively affected neurosphere cell growth as measured by the reduced number of cells obtained 7 days in the presence of OSM which is also illustrated by the decrease in MKi67^+^ cells (Figure 5G).

Last, we determined the influence of OSM during the differentiation phase of neurosphere cells into different cell types. Growing neurospheres were treated with OSM for 3 days and then for 3 additional days during the differentiation phase (w/o growth factors plus serum). Differentiation into astrocytes, oligodendrocytes and neurons was quantified by immunostainings for GFAP, OLIG1 and MAP2/TUBB3 respectively. In control condition, most neurosphere cells differentiated into astrocytes (approximately 80%) and no significant effect of OSM was observed on their production (Figure 5H). In contrast, OSM reduced by half the percentage of OLIG1^+^ cells indicating that this cytokine negatively impacted on the formation of oligodendrocytic cells (Figure 5H). With regard to the neuronal differentiation, the formation of MAP2^+^ or TUBB3^+^ neuronal cells was very weak (typically under 1%, data not shown) and highly variable between experiments and thus no reliable conclusion could be obtained.

All together, these results indicate that spinal cord neurosphere cells are highly responsive to OSM cytokine that alters their proliferation and differentiation into different cell types.

### 3.4. OSM Expression Is Strongly Upregulated in SCI

**Figure 6:**
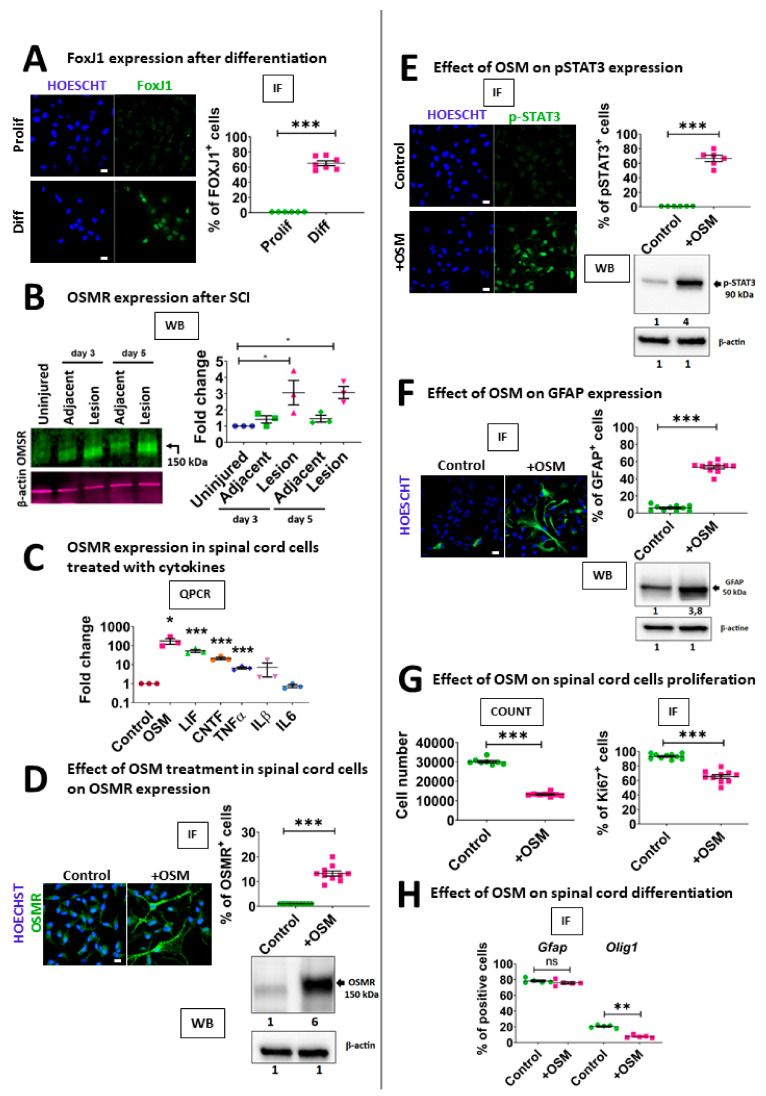
Microglia upregulate OSMR expression in spinal cord neurosphere cells. (**A**): QPCR for indicated cytokines in RNA extracted in sham-operated and injured spinal cord (3 days after SCI). *n* = 3 independent experiments. Numbers indicate the fold change compared to sham-operated spinal cords. Statistical tests were performed with Bootstratio (Clèries et al., 2012) compared to control spinal cords. (**B**): Immunofluorescences for GFP performed in CX3CR1^+/GFP^ mice to reveal microglia (green) associated with ependymal cells. White arrowheads and arrows show microglia somas and processes respectively, close or within the ependymal cell layer. Scale bars = 10 µm. (**C**): Left-hand panel: detection of the OSM cytokine in the supernatant of BV-2 microglial cell culture by ELISA (*n* = 4 independent experiments). Right-hand panel: detection of indicated cytokines in BV-2 microglia by QPCR (*n* = 3 independent experiments). (**D**): Influence of BV-2 cells on OSMR expression in spinal cord neurosphere cells. Left-hand panel: QPCR for OSMR. RNA were extracted from GFP^+^ spinal cord neurosphere cells cultured without (control) and with BV-2 cells. Values represent fold change compared to control neurospheres. Statistical test was performed with Bootstratio (Clèries et al., 2012) compared to control neurospheres. *n* = 3 independent experiments. Lower panel: Immunofluorescences for OSMR in GFP^+^ spinal cord neurosphere cells cultured without (control) or with BV-2 microglia. Scale bars = 10 µm. Right-hand panel: Quantification of immunofluorescences (*n* = 6 fields). test= two tailed *t*-test. (**E**): Influence of BV-2 cells on GFAP expression in spinal cord neurosphere cells. Left-hand panel: QPCR for GFAP. RNA were extracted from spinal cord neurosphere cells cultured without (control) and with BV-2 cells. Values represent fold change compared to control neurospheres. Statistical test was performed with Bootstratio (Clèries et al., 2012) compared to control neurospheres. *n* = 3 independent experiments. Lower panel: Immunofluorescences for GFAP in GFP^+^ spinal cord neurosphere cells cultured without (control) or with BV-2 microglia. Scale bars = 10 µm. Right-hand panel: Quantification of immunofluorescences (*n* = 10 fields). test= two tailed *t*-test. ***, *p* < 0.001; **, *p* < 0.01; *, *p* ≤ 0.05. n.s. = not significant. IF = immunofluorescence.

Considering the major effect of OSM on OSMR expression, proliferation and differentiation of spinal cord cells in vitro, we questioned the origin of this cytokine. Two brain cell-type-specific RNA databases indicated that Osm is almost exclusively expressed by microglia especially after their activation (Figure S2A,B). In addition, in a database comparing gene expression before and after thoracic contusive injury of the spinal cord Osm and the related cytokines Lif and Cntf are highly upregulated (Figure S2C) [51]. More recent single cells RNA seq analyses performed in a contusion model of SCI also reported highest expression of Osm in myeloid cells and its upregulation after injury [52]. To see how Osm expression varies in our model of SCI, we performed QPCR on total spinal cord RNA extracted before and 3 days post-injury and found that Osm is strongly upregulated after SCI (Figure 6A) (fold change: 5–10×, *n* = 3 mice). Expression of Cntf, Lif and Tnfa, which we showed can upregulate Osmr in neurosphere cells (Figure 5C), are also increased after SCI but more moderately than Osm (Figure 6A).

### 3.5. Microglia Upregulates Osmr in Spinal Cord Neurosphere Cells

Considering that microglia are the main source of Osm in SCI, we next investigated whether these cells could influence spinal cord ependymal cell fate. First we examined a possible physical interaction of these two cell types in the spinal cord especially as in the brain, microglia closely interacts with neural stem cells [53]. We used the CX3CR1^+/GFP^ transgenic mice to visualize microglia including their soma but also their processes as these cells are highly ramified. Figure 6B illustrates the presence of microglia soma (white arrowheads) in close contact with ependymal cells. Closer examinations also revealed microglia processes in the proximity of ependymal cells suggesting possible interactions (Figure 6B white arrows). Interactions between these two cell types can also be observed in the Gensat database [54] with two other microglial specific transgenic GFP mice (Limd2 and Csf2rb2) (Figure S3). Second, these observations encouraged us to investigate whether microglia could increase Osmr expression in spinal cord neurosphere cells. We based our approach on the mouse microglial BV-2 cell line [21]. Compared to primary microglial cells, this line immortalized by the v-raf/v-myc oncogenes showed differences in TGFβ signaling and chemotaxis [55,56] but remains extensively employed as a model for murine microglia [57,58]. Database screening indicated that BV-2 cells express Osm RNA at a level comparable to primary brain postnatal microglia (supplemental file 4 in [55]). To really ascertain the expression of Osm at the protein level in these cells, we used ELISA. Figure 6C showed that indeed, OSM was readily detected in the medium conditioned by BV-2 cells whereas this cytokine was undetectable in the medium conditioned by spinal cord neurosphere cultures. QPCR analysis also revealed that in addition to Osm, BV-2 cells express genes for other cytokines (Cntf, Lif and Tnfa) (Figure 6C right). Next, we tested the influence of BV-2 cells on Osmr expression by co-culturing neurosphere cells with these cells for 3 days. For this experiment, we used spinal cord neurospheres derived from the spinal cord of β-actin GFP transgenic mice which enabled us to sort these cells after co-culture. Osmr level was measured by QPCR. Results presented on Figure 6D indicates a strong upregulation (>10-fold) of Osmr after co-culture with BV-2 cells. This was validated by immunofluorescence, showing that OSMR is detected in a fraction of neurosphere cells after co-culture with BV-2 cells (Figure 6D right). We also found that co-culture with BV-2 cells strongly upregulate GFAP in neurosphere cells as evidenced by QPCR and immunofluorescence (Figure 6E). Collectively these results support the notion that microglia participate in the Osmr upregulation and astrocytic differentiation of spinal cord ependymal cells through the release of OSM.

## 4. Discussion

In this work, we built up a new corpus of knowledge on spinal cord ependymal cells, which are an important source to replace damaged cells after lesion. The reaction of ependymal cells to SCI has been observed since 1962 [11], however very little was known on the underlying molecular mechanisms. We addressed this key issue by studying the RNA profiles of ependymal cells during SCI. Three main conclusions can be drawn from this work.

First, pathway analysis and immunofluorescence demonstrated that SCI activates the ERK/MAPK and STAT3 pathways in a fraction of ependymal cells. What is the origin of these activations? Many molecules such as IL-6 related cytokines (involved in inflammation), growth factors (i.e., EGF) and even ECM proteins such as fibronectin [59] activate STAT3 and ERK/MAPK signalings. During SCI, the level of many of these proteins increases in the parenchyma [51] and in the ependyma itself (for instance fibronectin and the STAT-activating cytokine CLCF1 were upregulated 7x, Table S1). One possible candidate we identified is OSM but considering the variety of factors released during SCI, other molecules could also contribute to activating STAT3 and ERK/MAPK pathways. What are the consequences of these activations on ependymal cells? Both pathways can promote astrocytic differentiation of brain neural stem cells [37,48,60]. They may thus have similar effects in spinal cord especially as we observed that ependymal cells upregulated astrocyte-specific genes and downregulated ependymocyte-specific genes post injury. Genetic-tools have shown that ependyma-derived astrocytes have a positive effect on SCI [12] suggesting that STAT3 activation in ependymal cells is beneficial in this context. However, STAT3 acts a negative-regulator of neurogenesis in neural stem cells [61,62,63] which could be responsible for the absence of new neurons production by ependymal cells after injury. Besides differentiation and depending on their level of activation, ERK/MAPK and STAT3 pathways can also promote stem cell proliferation [63,64]. Thus they could also participate in the proliferation of ependymal cells induced by injury [11]. ERK and STAT3 pathways could also upregulate several ECM genes in ependymal cells. For instance, expression of Ecm1 and Vcan are strongly increased after SCI (Table 1), and these genes are controlled by ERK and STAT3 in other contexts [65,66]. These ECM proteins may help migration of central canal cells toward the lesion site and/or fill extracellular space left by cell death. Finally, activation of ERK and STAT3 pathways on ependymal cells likely acts through downstream transcription factors. Etv5 and Cebpd are potential candidates as their expression sharply rises after SCI (Table 1) and ERK and STAT3 pathways control their expression in other contexts [37,67]. Further investigations are required to understand the intimate and temporal crosstalks between these two pathways to regulate ependymal cell proliferation and differentiation.

A second significant and very unexpected result is that ependymal cells downregulate genes involved in ciliogenesis after SCI. As ciliogenesis opposes proliferation in many cell types, it is likely that this reduction in cilia-related genes reflects the mitotic reentry of ependymal cells. A recent study based on a single cell RNA seq approach of a contusion model of SCI also reported a reduction of cilia-related genes, including the master transcription factor of ciliogenesis (FoxJ1) in ependymal cells (Figures S4 and S5 in [52]). This illustrates the significance and robustness of this finding across different SCI models. Whereas Foxj1 was not significantly decreased transcriptionally in our study, we observed a reduction of the FOXJ1 protein in ependymal cells in vivo and also in vitro using spinal cord organotypic slices. This reduction could result from the degradation of FOXJ1 as this transcription factor has a short half-life and is constantly targeted by the ubiquitin-proteasome in brain ependymal cells [46]. Using primary spinal cord neurosphere cultures, we also observed that cilia-related genes and cilia transcription factors were expressed at a much lower level than in the spinal cord ependyma. Then, by removing and reintroducing EGF/FGF2 in the medium, we found that the expression level of a large fraction of these genes, notably Foxj1, increases and decreases respectively. This shows that these growth factors control ciliogenesis in spinal cord stem cells in vitro. This regulation of ciliogenesis is reminiscent of the situation observed in brain ependymal cells [46]. Here, upon growth factor stimulation in vitro, brain ependymal cells lose FoxJ1 expression and cilia, de-differentiate and proliferate. A similar scenario may apply in spinal cord ependymal cells but the growth factors triggering ependymal cells proliferation in vivo remain to be fully identified. The mechanisms suppressing ciliogenesis in dividing cells have been partly elucidated in cancer and ERK signaling is involved [68]. As this pathway is activated in ependymal cells after SCI, it appears as a good candidate to reduce ciliogenesis in these cells.

Finally, this work unveiled the OSM/OSMR pathway as part of the reaction of spinal cord ependymal cells to SCI. OSMR was the second top upregulated gene after SCI with a 30-fold increase, which we confirmed at the protein level in vivo and in vitro. Using spinal cord neurospheres, we identified this pathway as a regulator of spinal cord stem cell proliferation and astrocytic differentiation. Indeed, OSM reduced neurosphere cell proliferation and increased p-STAT3, OSMR and GFAP. OSM also reduced the fraction of OLIG1^+^ oligodendrocytic cells during differentiation. These results are consistent with the role of OSM/OSMR in other contexts. In brain, OSM reduces the proliferation of neural stem cells in vitro and their number in vivo [49] while promoting their astrocytic differentiation [69]. Besides, in various models OSM can induce both p-STAT3 and SOCS3 [70], a protein limiting STAT3 signaling and which is one of the top 10 gene upregulated after SCI (Table 1).

What triggers Osmr expression in ependymal cells after SCI? We found that OSM is a very potent inducer of Osmr expression in neurosphere cells. Transcriptomics databases revealed that Osm is almost exclusively expressed by microglia and its expression is rapidly and strongly increased after injury in brain (×20, [71]) and in spinal cord (×100 [72], ×20 [51], ×3 [52], ×5–10 this study). In vitro we found that a widely-used microglial cell line (BV-2) can upregulate OSMR in spinal cord neurospheres. Whereas this cell line presents some limitations [55,56], it expresses Osm gene at a comparable level to primary microglial cell cultures [55] and we detected production of OSM by these cells. We also observed close interactions between microglia and ependymal cells in vivo. Altogether, this suggests that microglia-derived OSM appears as a good candidate to induce OSMR expression in ependymal cells. Microglia could also contribute to spinal ependymal cell differentiation towards astrocytes in vivo as observed with brain stem cells [73,74] and as found in this study using spinal cord neurosphere cells. Nevertheless, we also observed that OSMR expression can be triggered by other cytokines such as LIF or CNTF that are expressed by reactive astrocytes in SCI [75] and literature shows that lipids such as prostaglandin E2 also increase OSMR [76]. Thus, in addition to OSM and microglia, several molecules and reactive astrocytes may also contribute to increasing OSMR expression in ependymal cells after injury which merits further investigation.

What is the significance of OSMR upregulation in spinal cord ependymal cells during injury? In vitro, we found that exposure of growing spinal cord neurosphere cells to OSM induced a sharp OSMR expression concomitantly with an astrocytic differentiation of these cells as evidenced by the expression of GFAP and p-STAT3. Also, addition of OSM during the neurosphere differentiation phase reduced the formation of oligodendrocyte cells. Thus, the observed upregulation of OSMR, GFAP and p-STAT3 in ependymal cells in vivo are suggestive of their commitment towards astrocytic differentiation may be at the expense of oligodendrocyte lineage cells.

How to reconcile the antiproliferative effect of OSM/OSMR signaling on ependymal cells we observed in vitro, together with its massive upregulation in these cells during the proliferation phase following SCI? It is possible that growth factors, yet to be identified, can trigger OSMR^+^ ependymal cells proliferation in vivo. Alternatively, a different temporal regulation of ERK and STAT3 pathways could successively induce proliferation then differentiation of ependymal cells.

One limitation of our study is that considering the cellular heterogeneity present in the ependymal region, the gene variations we identified may only apply to some cell subpopulations. Indeed, Crym, the top upregulated gene we detected after SCI in the ependymal region, was also recently identified in SCI through a single cell RNA seq approach [52]. In this contusion model of SCI, Crym was specifically upregulated in a new subpopulation of cells derived from ependymal cells called astroependymal cells. Another limitation is the use of in vitro models to study the role of oncostatin and microglial cells on the astrogenesis of spinal cord ependymal cells. Additional work performed in vivo with a targeted reduction of oncostatin in microglial cells is needed to validate the importance of these cells and this pathway in mediating the astrogenesis observed in ependymal cells following injury.

## 5. Conclusions

It remains unanswered why, compared to zebrafish, mammal ependymal cells generate mainly astrocytes, few oligodendrocytes, and no neurons after SCI. This could be due to astrocytic-fate determining factors expressed by these cells prior to injury such as SOX9 or NFIA transcription factors [6] or by structural proteins in the environment such as myelin basic protein [77]. In addition and reminiscent of the zebrafish situation [78], here we found evidence that their fate could be influenced by inflammatory cytokines provided by the environment, possibly microglia. Collectively, this work constitutes a molecular resource to further study ependymal cell response after SCI. This resource also sheds light on genes that are poorly annotated such as Tchh, Rgs20, and Gbp3 and which are highly deregulated during SCI in ependymal cells.

## Figures and Tables

**Table 1:** Main post injury up- and downregulated genes.

**Fold Change > 10**				
**Gene Symbol**	**Description**	**Fold Change**	***p*-Value**	**FDR**
*Crym*	crystallin. mu	33.3	1.6 × 10^−8^	2.5 × 10^−5^
*Osmr*	oncostatin M receptor	30.3	2.6 × 10^−10^	1.7 × 10^−6^
*Rbp1*	retinol binding protein 1. cellular	28.8	1.8 × 10^−7^	0.0001
*Nupr1*	nuclear protein transcription regulator 1	26	2.2 × 10^−6^	0.0006
*Thbs2*	thrombospondin 2	25.8	3.8 × 10^−12^	5.6 × 10^−8^
*Ecm1*	extracellular matrix protein 1	24.1	4.5 × 10^−10^	2.2 × 10^−6^
*Ifi202b*	interferon activated gene 202B;	22.8	2.6 × 10^−7^	0.0001
*Cebpd*	CCAAT/enhancer binding protein (C/EBP). delta	15.4	1.0 × 10^−5^	0.0017
*Timp1*	tissue inhibitor of metalloproteinase 1	15.3	8.1 × 10^−10^	2.7 × 10^−6^
*Socs3*	suppressor of cytokine signaling 3	14.4	5.1 × 10^−8^	5.2 × 10^−5^
*Olfml3*	olfactomedin-like 3	13.3	1.7 × 10^−10^	1.3 × 10^−6^
*Gfap*	glial fibrillary acidic protein	12.9	9.8 × 10^−7^	0.0004
*Fos*	FBJ osteosarcoma oncogene	12.7	1.1 × 10^−7^	8.4 × 10^−5^
*Etv5*	ets variant 5	12.6	3.1 × 10^−10^	1.8 × 10^−6^
*Serping1*	serine peptidase inhibitor. clade G. member 1	12.2	9.6 × 10^−9^	1.8 × 10^−5^
*Ptx3*	pentraxin related gene	12.2	9.4 × 10^−7^	0.0004
*Fgfrl1*	fibroblast growth factor receptor-like 1	11.9	5.5 × 10^−8^	5.5 × 10^−5^
*Vcan*	versican	11.6	2.9 × 10^−5^	0.003
*S100a6*	S100 calcium binding protein A6 (calcyclin)	11.3	0.0006	0.0265
*Serpina3n*	serine peptidase inhibitor. clade A. member 3N	11.3	4.2 × 10^−9^	1.1 × 10^−5^
*Gm42151*	predicted gene. 42151	11.1	8.2 × 10^−10^	2.7 × 10^−6^
*Lgals1*	lectin. galactose binding. soluble 1	11.1	2.0 × 10^−6^	0.0006
*Fgl2*	fibrinogen-like protein 2	10.7	1.4 × 10^−8^	2.2 10^−5^
*Gbp3*	guanylate binding protein 3	10.1	0.017	0.23
**Fold Change < −4**				
**Gene Symbol**	**Description**	**Fold Change**	***p*-Val** **ue**	**FDR**
*Acta1*	actin. alpha 1. skeletal muscle	−21.2	0.035	0.35
*Pcsk2*	proprotein convertase subtilisin/kexin type 2	−7.4	1.3 × 10^−6^	0.0004
*Snca*	synuclein. alpha	−5.8	0.0009	0.036
*Myh1*	myosin. heavy polypeptide 1. skeletal muscle. adult	−5.3	0.007	0.13
*Snca*	synuclein. alpha	−5.2	0.018	0.24
*Tchh*	trichohyalin	−4.7	0.0005	0.027
*Myh1*	myosin. heavy polypeptide 1. skeletal muscle. adult	−4.7	0.007	0.137
*Chd3os*	chromodomain helicase DNA binding protein 3. opposite strand	−4.6	1.5 × 10^−5^	0.0021
*Mb*	myoglobin	−4.6	0.0001	0.008
*Sema5a*	Semaphorin 5A	−4.4	0.015	0.22
*Snhg11*	small nucleolar RNA host gene 11	−4.4	0.006	0.11
*Ccdc108*	coiled-coil domain containing 108	−4.2	3.5 × 10^−6^	0.0008
*Tnni2*	troponin I. skeletal. fast 2	−4.1	0.02	0.254
*Plppr4*	phospholipid phosphatase related 4	−4.1	3.3 × 10^−5^	0.004
*Clstn2*	calsyntenin 2	−4.1	0.002	0.05
*Tnni3*	troponin I. cardiac 3	−4.0	6.6 × 10^−5^	0.006
*Acsl3*	acyl-CoA synthetase long-chain family member 3	−4.0	0.019	0.25

## Data Availability

The transcriptomic raw data that support the findings of this study are openly available at the functional genomics data Gene Expression Omnibus (GEO: GSE149669).

## Supplementary Materials

The following are available online at www.mdpi.com/article/10.3390/cells10123332/s1, Figure S1: Transcriptome data analysis. Figure S2: OSM expression in normal and injured CNS. Figure S3: Database images of microglia around the spinal cord central canal region. Table S1: detailed analysis of the RNA profiles of ependyma cells before and after SCI. Table S2: primers and antibodies used in the study.
