# Supplementary material for: RNA Profiling of Mouse Ependymal Cells after Spinal Cord Injury Identifies the Oncostatin Pathway as a Potential Key Regulator of Spinal Cord Stem Cell Fate
Source: Cells. 2021 Nov 27;10(12):3332. doi: 10.3390/cells10123332 (PMC8699053; doi:10.3390/cells10123332)
Supplement: Supplementary file 1 [file cells-10-03332-s001.zip › Supplemental Figures and Data/Supplementary figures S1-3.pdf]

FIGURE S1

A

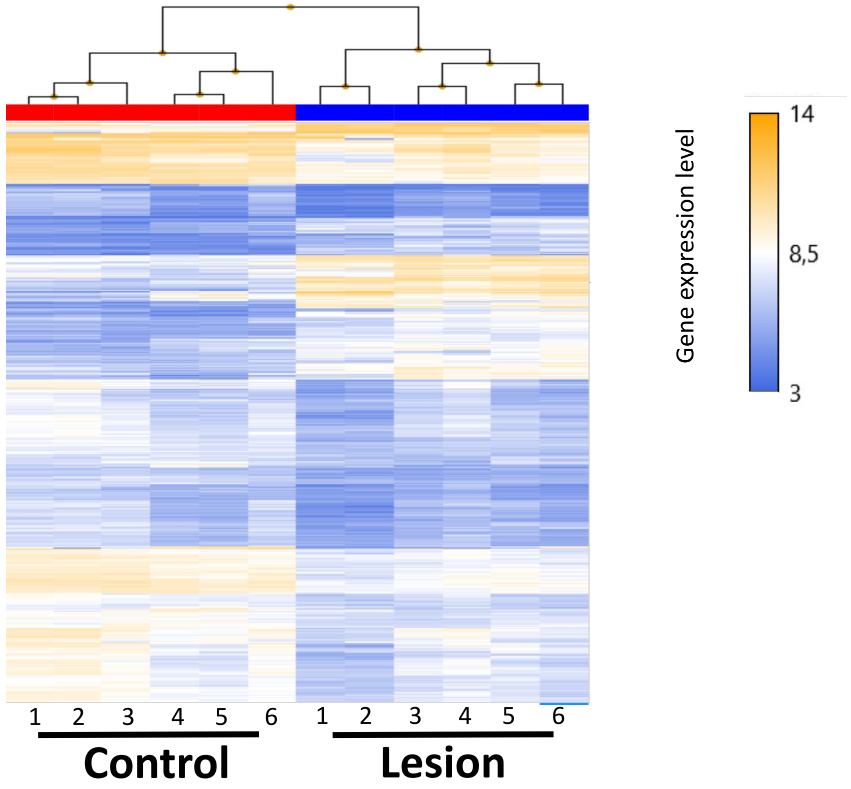

B

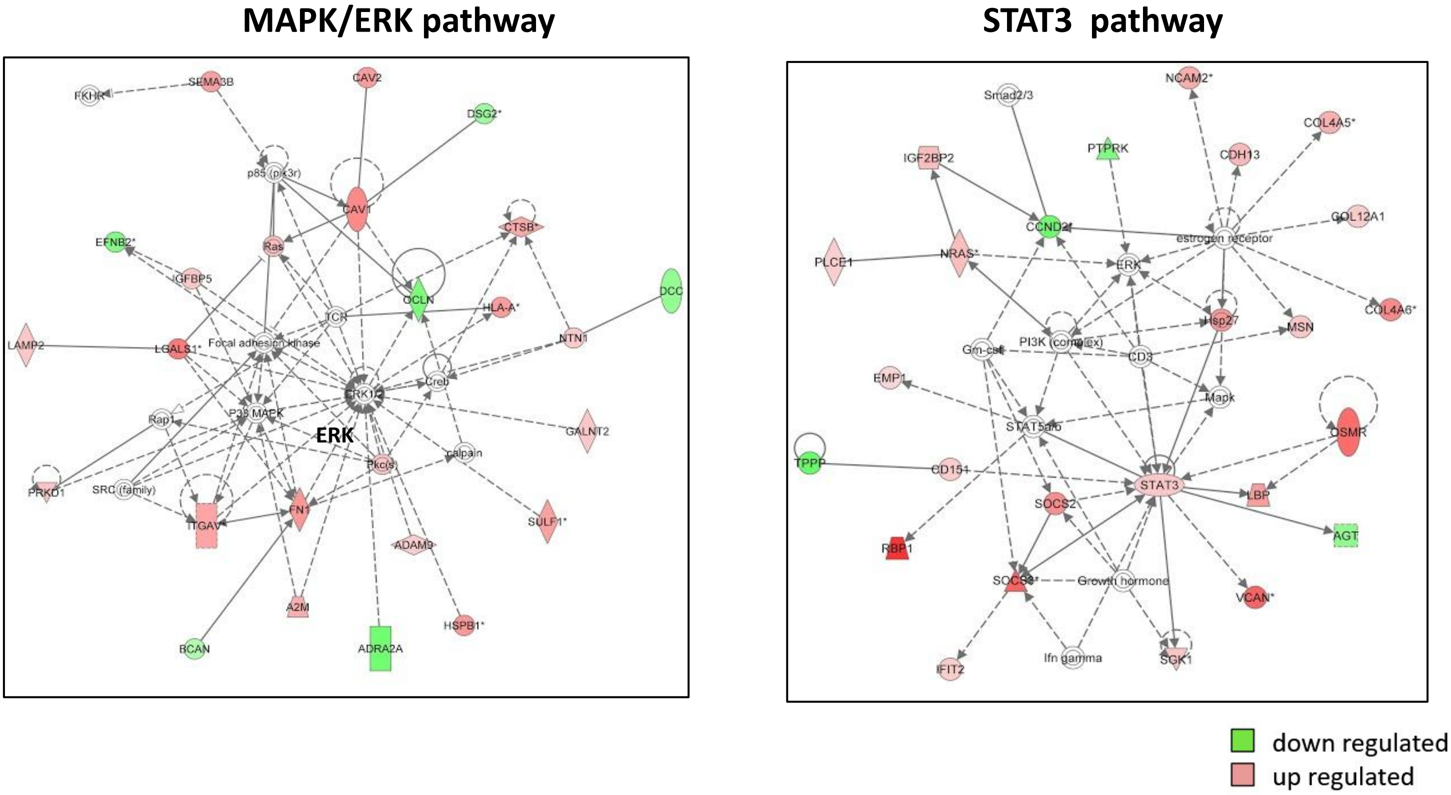

Figure S1: Data analysis. A: Heatmap of hierarchical clustering of genes differentially expressed in the ependymal region (control vs injured, p value ≤0.05, fold change≥2 or ≤-2, 1226 genes, n=6 mice per group). Sample numbers are indicated under the diagrams. **B:** Ingenuity pathway analysis of differentially expressed genes.

FIGURE S2

A

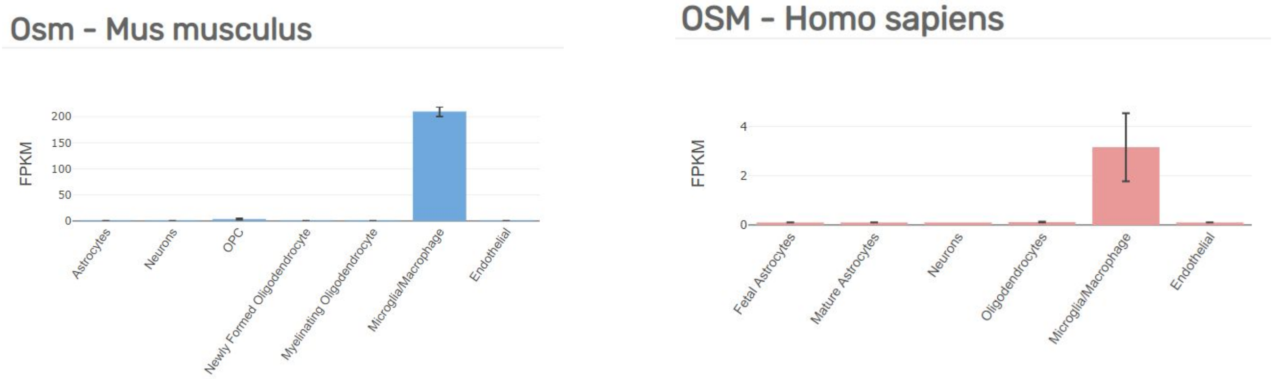

B

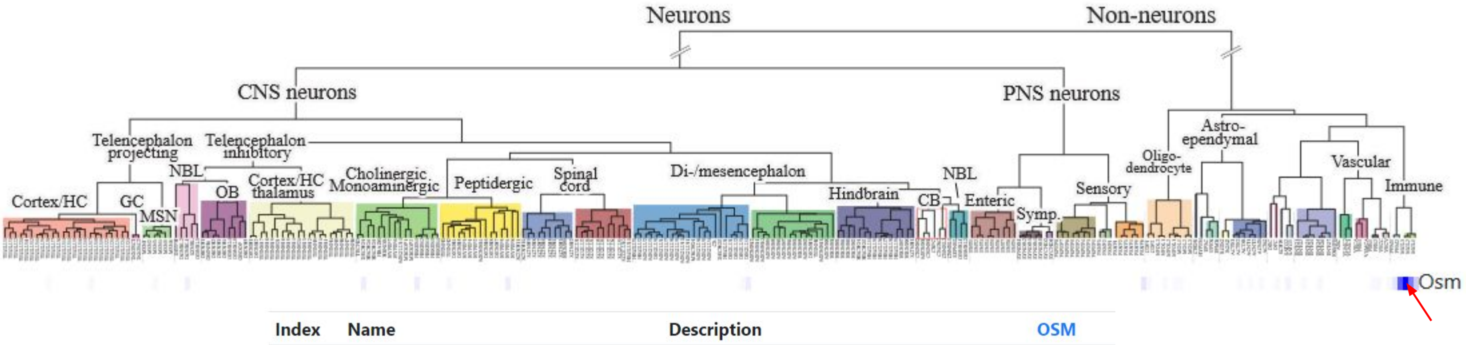

Index	Name	Description	OSM
261	PVM1	Perivascular macrophages	0.0139
262	PVM2	Perivascular macrophages, activated	0.135
263	MGL3	Microglia, activated	0.210
264	MGL2	Microglia, activated	0.104
265	MGL1	Microglia	0.0381

C

RNA levels after SCI

(adapted from data published in Chen K et al, PLoS One. 2013 Aug 9;8(8):e72567)

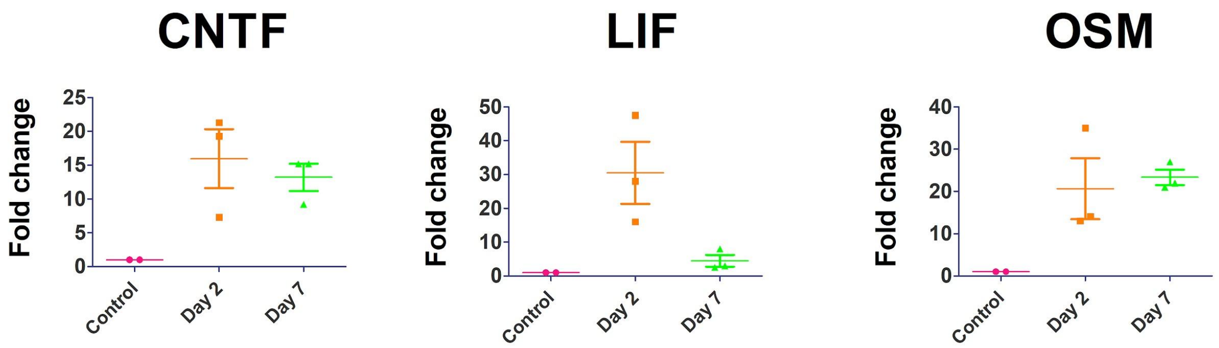

Figure S2: OSM expression in normal and injured CNS. A: In human and mouse CNS atlas (Brain RNA seq database Ben Barres's lab) OSM is specifically expressed in microglial cells. These images are derived from the database (brainrnaseq.org) associated to this publication (Zhang, et al. Neuron (2016) Jan 6;89(1):37-53). **B**: *Upper panel*: Single cell RNA expression confirms that OSM is mainly expressed by immune cells (microglia and macrophage) in the mouse CNS. These data were obtained from the database (mousebrain.org) associated to this publication (Zeisel et al, Cell. (2018) Aug 9;174(4):999-1014). Adult cell subtypes showing OSM expression are indicated by a blue rectangle under the hierarchical tree. Red arrow points to the microglia and macrophage subpopulations. *Lower panel*: Quantitative expression of OSM in these cells is presented as a blue circle and underneath value. **C:** Expression of *Cntf*, *Lif* and *Osm* cytokines by RNA seq after acute spinal cord injury. Data represent fold change compared to non injured spinal cord (control). These data were plotted after extraction from the database associated to this publication (Chen K et al, PloS One. (2013) Aug 9;8(8):e72567)

FIGURE S3

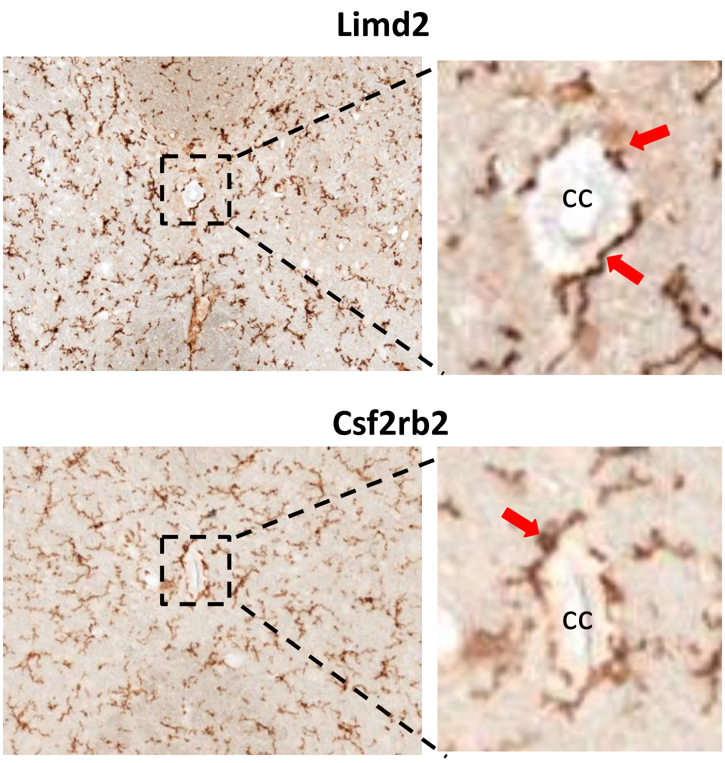

Figure S3: Microglia around the spinal cord central canal region. Images imported from the Gensat expression database (gensat.org, Heintz N et al, (2004), Nat Neurosci. May;7(5):483) for 2 microglia specific genes (Limd2 and Csf2rb2). Brown stainings are IHC for GFP protein expressed under the control of Limd2 and Csf2rb2 promoters. Right-hand images are high magnification of the central canal region (cc). Red arrows indicate microglial processes surrounding the ependymal region.
