# Supplementary material for: RNA Profiling of Mouse Ependymal Cells after Spinal Cord Injury Identifies the Oncostatin Pathway as a Potential Key Regulator of Spinal Cord Stem Cell Fate
Source: Cells. 2021 Nov 27;10(12):3332. doi: 10.3390/cells10123332 (PMC8699053; doi:10.3390/cells10123332)
Supplement: Supplementary file 1 [file cells-10-03332-s001.zip › Supplemental Figures and Data/Tables S2 Antibodies and primers.pdf]

Table S2
Antibodies

Name	*Specie*	*Supplier*	*Reference*	*Dilution for IF*	*Dilution for WB*
**β-ACTIN**	Mouse	Cell Signaling Technology	8H10D10	N.A.	1/1000
**FOXJ1**	Mouse	eBioscience	14-9965-82	1/100	N.A.
**GFAP**	Rabbit	Dako	Z0334	1/2000	1/5000
**GFP**	Rabbit	Abcam	ab183734	1/500	N.A.
**KI67**	Mouse	Pharmingen	556003	1/500	
**NETRIN**	Rabbit	Abcam	ab126729	1/500	N.A.
**OLIG1**	Goat	R&D	AF2417	1/100	N.A.
**OSMR**	Goat	R&D	AF662-SP	1/500	1/1000
**p-ERK**	Rabbit	Cell Signaling Technology	4370	1/200	N.A.
**p-STAT3**	Rabbit	Cell Signaling Technology	#9131	1/500	1/250
**TAG** (DYKDDDDK)	Mouse	Supernatant	HB-9259	1/500	N.A.

Primers

Name	***Specie***	***Forward***	***Reverse***	***Size***
b-Actin	Mouse	GGCTGTATTCCCCTCCATCG	CCAGTTGGTAACAATGCCATGT	154
Cntf	Mouse	GGTGACTTCCATCAGGCAAT	CCATCAGCCTCTTTTTCAGG	116
Lif	Mouse	AGAAGGTCCTGAACCCCACT	CCACACGGTACTTGTTGCAC	117
Osm	Mouse	TCAGGGGTCTGATGACACAA	GAGAGAGCACCGGATCAGAC	160
Osmr	Mouse	CATCCCGAAGCGAAGTCTTGG	GGCTGGGACAGTCCATTCTAAA	110
Tnfa	Mouse	AGGGGATTATGGCTCAGGGT	GAGTCCTTGATGGTGGTGCA	120
